# Probing magnetism in atomically thin semiconducting PtSe_2_

**DOI:** 10.1038/s41467-020-18521-6

**Published:** 2020-09-23

**Authors:** Ahmet Avsar, Cheol-Yeon Cheon, Michele Pizzochero, Mukesh Tripathi, Alberto Ciarrocchi, Oleg V. Yazyev, Andras Kis

**Affiliations:** 1 Electrical Engineering Institute, École Polytechnique Fédérale de Lausanne (EPFL), Lausanne, 1015 Switzerland; 2 Institute of Materials Science and Engineering, École Polytechnique Fédérale de Lausanne (EPFL), Lausanne, CH 1015 Switzerland; 3 Institute of Physics, École Polytechnique Fédérale de Lausanne (EPFL), Lausanne, CH 1015 Switzerland

**Keywords:** Two-dimensional materials, Condensed-matter physics

## Abstract

Atomic-scale disorder in two-dimensional transition metal dichalcogenides is often accompanied by local magnetic moments, which can conceivably induce long-range magnetic ordering into intrinsically non-magnetic materials. Here, we demonstrate the signature of long-range magnetic orderings in defective mono- and bi-layer semiconducting PtSe_2_ by performing magnetoresistance measurements under both lateral and vertical measurement configurations. As the material is thinned down from bi- to mono-layer thickness, we observe a ferromagnetic-to-antiferromagnetic crossover, a behavior which is opposite to the one observed in the prototypical 2D magnet CrI_3_. Our first-principles calculations, supported by aberration-corrected transmission electron microscopy imaging of point defects, associate this transition to the interplay between the defect-induced magnetism and the interlayer interactions in PtSe_2_. Furthermore, we show that graphene can be effectively used to probe the magnetization of adjacent semiconducting PtSe_2_. Our findings in an ultimately scaled monolayer system lay the foundation for atom-by-atom engineering of magnetism in otherwise non-magnetic 2D materials.

## Abstract (web summary)

Beneficiary defects could be utilized to introduce magnetism into materials that are not intrinsically magnetic. Here, the authors demonstrate long range magnetic order in the air-stable, defective Platinum Diselenide in the ultimate limit of thickness by using proximitized graphene as a probe.

## Introduction

Two-dimensional (2D) transition metal dichalcogenides (TMDCs)^1^ are emerging as an appealing class of materials for a wide range of research topics^1^, including electronics^2^, spintronics^3–6^, and magnetism^7–10^. Among this rapidly expanding family of materials, layered platinum diselenide (PtSe_2_) is a relatively new and interesting member due to its unique thickness-dependent electronic structure^11–17^. While multilayer films exhibit metallic character, this crystal undergoes a metal-to-semiconductor transition when thinned down to mono- and bilayers^11–13^. Theoretical work has also indicated that electronic properties of such thin PtSe_2_ crystal can be further tuned by defect engineering^18^.

Recent experimental investigations highlighted a broad variety of intrinsic point defects in PtSe_2_^19^_._ Even though imperfections are often regarded as detrimental to material properties, in many cases their presence has proven successful in introducing functionalities that would otherwise be absent in pristine materials^20,21^. This is especially true in the case of metallic PtSe_2_ multilayers, where surface Pt vacancies trigger a thickness-dependent surface magnetic ordering^18,20^. The strength of the magnetic exchange coupling across the metallic multilayers is governed by the RKKY interactions. However, thin films of PtSe_2_ acquire a semiconducting character when approaching the single-layer regime^11,13^, and the physical picture governing the magnetic exchange interactions in the thinnest films of PtSe_2_ would rather invoke the direct and superexchange mechanisms, similarly to dilute magnetic semiconductors^22^. Understanding and controlling the interplay between disorder and magnetism in this ultimate thickness scale can spark unexplored directions for designing 2D semiconducting magnets through defect engineering^8^. In this Letter, we demonstrate that defect-induced magnetism is preserved in mono and bilayers of semiconducting PtSe_2_, with a monolayer, surprisingly, showing antiferromagnetic ordering, while bilayer PtSe_2_ is a ferromagnet.

## Results

### Device characterization

**Fig. 1:**
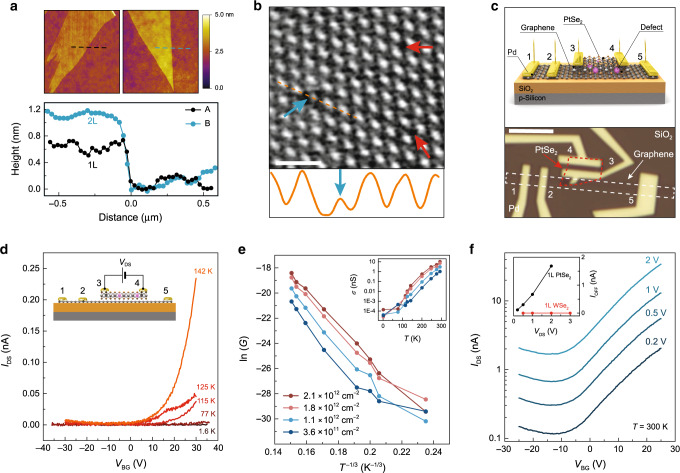
Device structure and basic characterization. **a** AFM images for mono- and bilayer PtSe_2_ which are utilized as active channel materials in characterized devices. Color scale of the AFM image is 0–5 nm. Following the dashed lines, a height of ~0.65 nm (~1.2 nm) is measured for monolayer (bilayer) PtSe_2_. Scan size: ~3 µm × 3 µm. **b** Raw HAADF image of a bilayer PtSe_2_. Blue and red arrows show the sites with missing Pt and Se atoms, respectively. Scale bar is 1 nm. Bottom panel shows the intensity profile measured along the dashed orange line shown in the STEM image. Blue arrow shows the intensity recorded at the Pt vacancy site. **c** Top panel: device schematics. A few layer thick PtSe_2_ with intrinsic defects is transferred on top of monolayer graphene. Both graphene and PtSe_2_ are contacted with non-magnetic Pd contacts (80-nm thick). Bottom panel: optical image of a completed device. Scale bar is 6 µm. **d** *V*_BG_ dependence of *I*_DS_ measured at fixed temperature values. *V*_DS_ = 2 V. Inset shows the device schematic. **e** ln(*G*) vs *T*^−1/3^ measured at different doping levels. Inset shows the temperature dependence of device conductance at different doping values. **f** Room-temperature *V*_BG_ dependence of *I*_DS_ measured at fixed *V*_DS_ values. Inset compares the *V*_DS_ dependence of measured off*-*state conductance for monolayer PtSe_2_ and WSe_2_ prepared under the same conditions.

Our ultra-thin PtSe_2_ flakes were obtained by mechanical exfoliation from CVT-grown bulk crystals (HQ Graphene), and their thicknesses were determined by atomic force microscopy (AFM). As shown in Fig. 1a, the thickness of a monolayer (bilayer) is found to be ~0.65 nm (~1.2 nm). Next, the mono- or bilayer crystal was transferred onto a monolayer graphene ribbon which was initially deposited onto a Si/SiO_2_ substrate. Graphene is utilized as a bottom electrode, thereby taking advantage of its electrostatic transparency. This enables gate-tunable vertical and lateral transport operations, as the screening length exceeds its monolayer thickness^23^. By employing electron-beam lithography and electron-beam evaporation techniques, non-magnetic metallic palladium (Pd) contacts (80-nm thick) were formed on top of PtSe_2_ and graphene. Further details concerning the device fabrication process are given in “Methods” section. The choice of non-magnetic graphene and Pd contacts rules out any influence of the electrodes on the magnetic response of the device. The schematics of the resulting device geometry is shown in Fig. 1c. This geometry allows us to probe the magnetism in PtSe_2_ through vertical transport by passing the charge current between electrodes #2 (graphene) and #3 (Pd). In addition, it allows us to characterize the basic transport properties of monolayer PtSe_2_ by utilizing Pd electrodes #3 and #4 in the lateral measurement geometry. We further compare the magneto-transport properties of isolated graphene as well as the graphene/monolayer PtSe_2_ heterostructure by relying on electrode pairs #1 & #2 and #2 & #5, respectively. An optical image of the completed device is presented in the bottom panel of Fig. 1c. The transport output was characterized as a function of back-gate voltage (*V*_BG_), source-drain bias (*V*_DS_), magnetic field, and temperature. Magnetic field-dependent measurements were performed at the base temperature (1.6 K) to maximize the signal-to-noise ratio.

### Charge transport in monolayer PtSe_2_

Prior to magnetic field-dependent measurements, we first assess the basic charge transport properties of monolayer PtSe_2_. Figure 1d shows the *V*_BG_ dependence of *I*_DS_ measured at temperatures covering the 1.6–142 K range, while maintaining a constant *V*_DS_ = 2 V. While *I*_DS_ is slightly modulated by the application of *V*_BG_ due to the semiconducting nature of monolayer PtSe_2_, the device shows a relatively low on-state conductance (*V*_BG_ > −5 V). The insulating nature of the crystal is unambiguously confirmed by the observed temperature dependence of the conductivity (*σ*) measured at fixed carrier concentrations (Fig. 1e-Inset). In the presence of disorder, the electrical transport can be described using the Mott formalism and the variable range hopping (VRH) model which describes the conductivity σ in terms of a characteristic temperature^24^. The observed linear dependence of the ln(*G*) on *T*^−1/3^ at different given carrier densities (see Fig. 1e and Supplementary Note 1) supports the 2D VRH nature of charge transport up to room temperature (RT). Such a characteristic temperature dependence has been widely observed in many systems, including defective 2D TMDC monolayers^25–27^. In fact, a bandgap of 1.2 eV is expected in monolayer PtSe_2_^12^ which in turn should result in a stable off state over a large *V*_BG_ range even at RT. For example, transport characteristics of monolayer black phosophorus^28^ and multilayer MoSe_2_^29^, which display comparable bandgap values with monolayer PtSe_2_, exhibit a stable off-state over 60 V when measured in a back-gate device geometry with an equivalent SiO_2_ thickness of ~270 nm. As shown in Fig. 1f, the lack of a clear off*-*state along with a strong dependence of the off-current on *V*_DS_ at RT signals the hopping nature of charge transport, and, importantly, further suggests the presence of atomic defects in monolayer PtSe_2_^27^.

### Imaging PtSe_2_ defects using transmission electron microscopy

In order to directly visualize the type of defects in our samples, we relied on Cs-corrected high-resolution transmission electron microscopy imaging. The result obtained from a bilayer PtSe_2_ with a low accelerating voltage of 80 kV is shown in Fig. 1b. Consistent with early scanning tunneling microscopy investigations^19^, we observe native Se and Pt vacancies in ultra-thin PtSe_2_ films. The concentration of Pt vacancy defects is found to be ~9 × 10^12^ cm^−2^ (see Supplementary Note 2). Similarly to the bilayer case, we also observe Pt vacancies in monolayers (see Supplementary Note 3).

### Magnetoresistance measurement in monolayer PtSe_2_

**Fig. 2:**
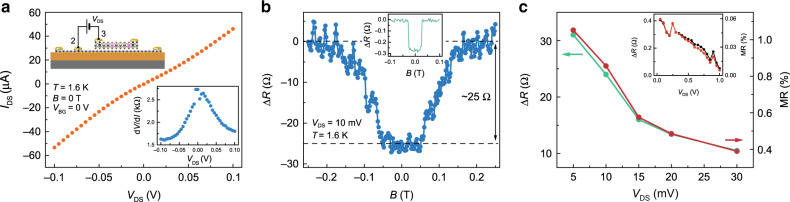
Vertical transport across monolayer PtSe_2_. **a** Bias voltage dependence of *I*_DS_. Top inset is the schematic of the measurement geometry. Bottom inset is the differential resistance as a function of applied bias. **b** Magnetic field dependence of electrical resistance measured at *T* = 1.6 K. The observation of a two-plateau response indicates the presence of antiferromagnetic ground-state ordering. Inset shows the magneto-transport measurements for a ~7-nm-thick metallic PtSe_2_. **c** Bias dependence of the change in device resistance as a result of the magnetic field and corresponding MR percentage. All measurements are taken at 1.6 K. Inset shows the bias dependence of resistance and MR for a ~7-nm-thick metallic PtSe_2_.

After confirming the presence of intrinsic defects in our crystals, we next study the magneto-transport characteristics of our monolayer PtSe_2_ sandwiched between the source (graphene) and drain (Pd) electrodes (inset of Fig. 2a) by utilizing a vertical measurement geometry. A fixed *V*_DS_ is applied to PtSe_2_ while measuring the dependence of device resistance on the applied magnetic field. As shown in Fig. 2a, the *I*_DS_–*V*_DS_ curve is nearly linear and symmetric, measured at zero field. As compared to the lateral transport measurement shown in Fig. 1d, the device resistance under vertical geometry is improved by several orders of magnitude, as a result of the reduced transport pathway. Such a highly conductive response obtained along the vertical direction allows us to probe directly the magnetic ordering of monolayer PtSe_2_. To this end, we sweep the out-of-plane magnetic field from −0.2 to 0.2 T while recording the vertical device resistance at *V*_DS_ = 10 mV. As clearly seen in Fig. 2b, the evolution of the resistance in our device with the magnetic field substantially departs from the typical localization response. Indeed, it features plateaus with two different values. As the field is increased, the lower plateau observed in the −50 mT < *B* < 50 mT interval is found to slowly approach the high plateau observed above 150 mT. This two-plateau response is the signature of an antiferromagnetic ground-state ordering^20,30,31^. While we find a similar two-plateau response in thicker films of PtSe_2_ (inset of Fig. 2b), the observation of a slow low-to-high plateau switching in monolayer PtSe_2_ indicates that the magnetic moments are not fully oriented along the out-of-plane direction, with spins that could be slightly canted. Similarly to metallic films, hysteresis in monolayer PtSe_2_ is almost absent (see Supplementary Note 4). Furthermore, the magnetoresistance (MR) percentage is found to be bias-dependent as in thicker metallic devices and estimated to be 1% in the low-bias regime (Fig. 2c).

**Fig. 3:**
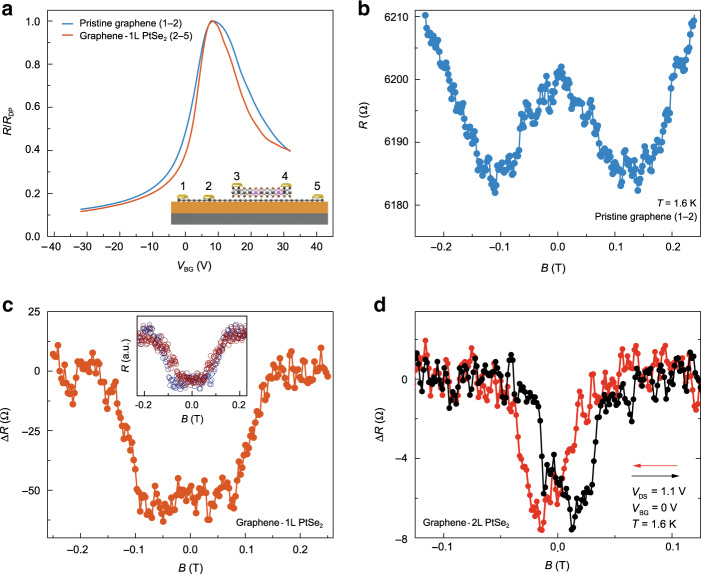
Induced magnetization in monolayer graphene. **a** *V*_BG_ dependence of graphene resistance. Red (blue) line represents the device with (without) a monolayer PtSe_2_ on top of the graphene channel, between the voltage probes. Inset is the schematics of the measurement geometry. **b** Magnetic field dependence of pristine graphene resistance measured at *T* = 1.6 K. **c** Magnetic field dependence of graphene resistance measured at *T* = 1.6 K for the device with monolayer PtSe_2_ on the channel. Inset shows the magnetic field response of vertically measured monolayer PtSe_2_ (red circles) and laterally measured graphene/monolayer PtSe_2_ (blue circles). They have similar out-of-plane coercive fields. **d** Magnetic field dependence of graphene resistance measured at *T* = 1.6 K for the device with a bilayer PtSe_2_ on the graphene channel. Arrows represent magnetic field sweep directions.

In order to investigate the impact of magnetic ordering in PtSe_2_ on the transport properties of graphene, we characterize the graphene device resistance with and without PtSe_2_ on the channel connecting the source and drain electrodes. Figure 3a displays the *V*_BG_ dependences of corresponding device resistances which show nearly identical device characteristics. However, the magnetic field responses of these junctions present striking differences, as shown in Fig. 3b, c. While the pristine graphene junction exhibits a conventional weak localization response^32^, the junction with the PtSe_2_ monolayer exhibits plateaus with two different values^20,30,31^. This difference in the magneto-transport properties is clearly due to the presence of magnetism in PtSe_2_ and could be ascribed to two possible origins. One possibility is the back-and-forth hopping of graphene’s electrons through the magnetic defect states in PtSe_2_ via quantum tunneling, a mechanism similar to the previously reported one in graphene/WS_2_ heterostructures^33^. This possibility is indeed supported by the observation of linear *I*_DS_–*V*_DS_ relation and the nearly identical shapes of the magnetic response of pure PtSe_2_ and the graphene/PtSe_2_ heterostructure, as shown in the inset of Fig. 3c. On the other hand, there could be contribution from the interface-induced magnetism^34^ by PtSe_2_.

### Magnetoresistance measurement in bilayer PtSe_2_

Finally, we complement our investigation by replacing monolayer PtSe_2_ with bilayer PtSe_2_. We observe a hysteresis loop with minima at ±15 mT under backward and forward scans (Fig. 3d), which is the hallmark of ferromagnetism^35^. The maximum change in the device resistance upon the application of a magnetic field is ~16 Ω and it strongly depends on the applied *V*_DS_. The MR percentage probed by graphene is ~0.1% and is nearly independent of the applied *V*_BG_ (see Supplementary Notes 5–6). Our findings suggest that monolayer (bilayer) PtSe_2_ exhibits long-range antiferromagnetic (ferromagnetic) ground-state ordering, and that adjacent graphene electrodes can effectively be used as a sensitive probe to detect such magnetism^36^. To shed more light on this antiferromagnetic-to-ferromagnetic crossover, measurement techniques such as single-spin microscopy^37^ can be employed at the nanoscale in future experiments. Such direct measurements would unambiguously determine the types of magnetism and its strength, as recently utilized for another layered magnet^37^.

Long-range magnetic ordering in semiconductors can be achieved through the incorporation of extrinsic magnetic impurities (e.g., Mn-doped GaAs^38^ and V-doped WSe_2_^39^). We rule out this possibility in our work by performing quantitative and qualitative assessment of our samples, which do not show the presence of any chemical elements except for Pt and Se and other crystal phases beside 1 T (see Supplementary Notes 7 and 8). Therefore, our findings constitute a model study demonstrating the purely defect-induced magnetism into an otherwise non-magnetic crystal down to the ultimately scaled monolayer system.

### First-principles calculations

**Fig. 4:**
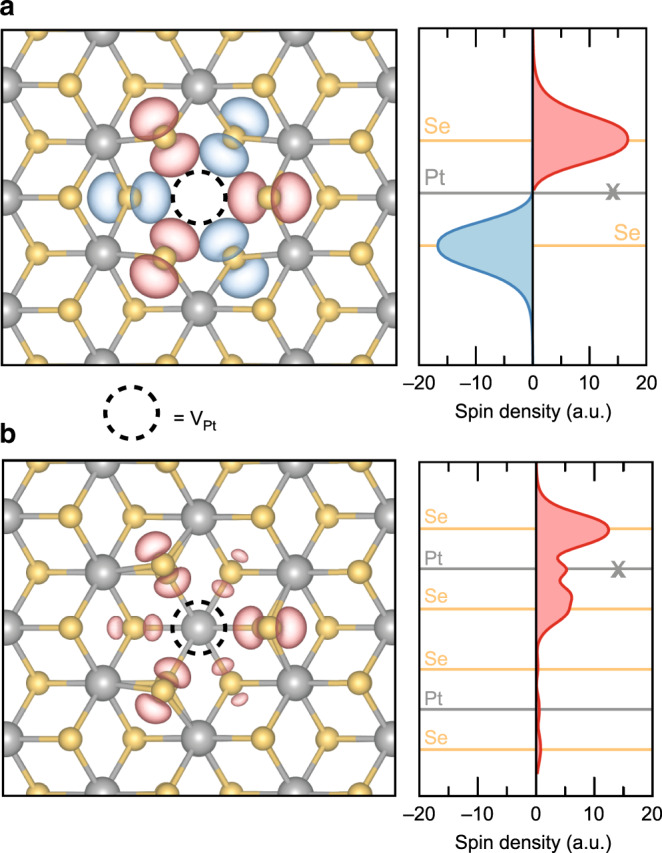
Localized magnetic moments at Pt vacancy defects in PtSe_2_. **a** Spin-density distribution at the ground-state configuration of the V_Pt_ defect in monolayer PtSe_2_ calculated from first principles. Gray and orange balls represent Pt and Se atoms, respectively. The red and blue isosurfaces correspond to positive and negative spin densities. The position of the missing Pt atom is marked with a dashed circle. The right panel shows the in-plane averaged spin density as a function of the out-of-plane position. The cross (X) indicates the Pt atomic plane at which the vacancy defect is introduced. **b** Spin-density distribution at the Pt vacancy in the top layer of bilayer PtSe_2_ calculated from first principles. Units in panels (**a**) and (**b**) agree with each other.

We now address the physical mechanism underlying the observed effects from a theoretical perspective. According to a purely ionic argument, Pt atoms exhibit a formal oxidation state of +4 and a resulting valence electron configuration 5d^6^6s^0^. On the basis of the crystal and ligand fields associated with the octahedral environment in which each Pt atom resides, a splitting of the d orbitals into a pair of empty e_2g_ states and fully occupied t_2g_ states is expected. Such a full occupation of the t_2g_ manifold prevents any intrinsic magnetism in PtSe_2_ at all possible thicknesses, from monolayer to bulk, as further confirmed by our explicit density-functional calculations. As in the case of metallic PtSe_2_^20^, we conclude that lattice imperfections are the origin of the observed layer-dependent magnetism in semiconducting PtSe_2_. Motivated by direct observation of Pt vacancies in our transmission electron microscope images shown in Fig. 1b, their indirect inference from our charge transport measurements as well as earlier scanning tunneling microscopy studies^19^, we investigate electronic and magnetic properties of mono- and bilayer models of PtSe_2_ with platinum vacancy defects by means of first-principles calculations. For the monolayer case, we find that a Pt vacancy defect in its fully spin-polarized configuration gives rise to a local magnetic moment of 4.00 μ_B_, which is localized mostly on the six selenium atoms neighboring to the missing Pt atom. We note, however, that the configuration characterized by local magnetic moments in the two Se atomic planes that are oriented antiparallel to each other (Fig. 4a) is 35 meV lower in energy than the former parallel configuration. Below, we will refer to this configuration with zero net magnetic moment as antiferromagnetic. Here, we would like to also note that the Se point defect does not lead to magnetism.

Next, we consider the bilayer PtSe_2_ model with Pt vacancy in one of the two layers. In contrast to the monolayer case discussed above, we could not identify any solution for a defective bilayer other than the ferromagnetic configuration with a net magnetic moment of 1.33 μ_B_ (Fig. 4b). This value agrees well with the one obtained for Pt vacancy defects in thicker metallic PtSe_2_ slabs^20^. The reason for this traces back to the existence of interlayer coupling between the PtSe_2_ sheets in the bilayer system. In our previous work^20^, we have shown that platinum vacancy defects located at the surfaces of multilayer PtSe_2_ act as magnetic centers, while their presence in the inner regions of the thick samples (as well as in the three-dimensional bulk material) does not lead to any local magnetic moment. This observation clearly pinpoints the pivotal role of interlayer interactions in quenching the defect-induced magnetic response in PtSe_2_. In bilayer PtSe_2_, the interlayer coupling affects only one of the two selenium atomic planes. As a result, the defect-induced magnetic moment in this system is reduced (1.33 μ_B_) as compared to the fully polarized magnetic moment of defect in monolayer PtSe_2_ (4.00 μ_B_), and, remarkably, the spin density primarily resides on the outmost selenium atoms in the defect (Fig. 4b). Overall, our first-principles calculations suggest that a platinum vacancy defect yields an antiferro- (ferro-)magnetic ordering in mono (bi-)layer PtSe_2_, thereby portraying a theoretical picture in accord with our experimental observations.

## Discussion

Antiferromagnets have been recently considered for sensing and memory applications^40^. For this purpose, layered intralayer antiferromagnetic compounds such as MnPS_3_, FePS_3_, and CoPS_3_ indeed show promise^9^. Among these, only a few of them have been exfoliated down to the monolayer. While antiferromagnetic ordering at the monolayer limit still persists in FePS_3_ with an unchanged transition temperature^41^, such ordering is suppressed in NiPS_3_^42^. Unlike FePS_3_, the observed antiferromagnetic ordering in monolayer PtSe_2_ is not intrinsic, rather originates from native impurities hosted in the material. Therefore, our work ignites the potential of atomic-scale defects for driving non-magnetic 2D crystals into magnetic phases, thereby opening new directions for expanding the library of two-dimensional magnets^43^.

## Methods

### Device fabrication

Our device fabrication starts with the mechanical exfoliation of monolayer graphene nanoribbons onto a doped Si substrate with 270 nm of SiO_2_. PtSe_2_ crystals are similarly obtained by the mechanical exfoliation from bulk crystals (HQ Graphene) onto a Si/SiO_2_ wafer. The thickness of selected crystals was determined by using AFM topography imaging (Asylum Research Cypher). PtSe_2_ was then isolated from its Si/SiO_2_ substrate with the help of a supporting PMMA layer by etching SiO_2_ via a diluted potassium hydroxide (KOH) solution (0.7%, ~3 h). This was followed by rinsing the isolated film in deionized (DI) water for several times prior to its transfer onto previously exfoliated graphene nanoribbons. After removing the supporting PMMA via a hot acetone treatment (~90 °C for 1 h) followed by cleaning with IPA. The resulting graphene/PtSe_2_ heterostructure was annealed at 250 °C for 6 h under high vacuum conditions (~1 × 10^−6^ mbar) to further remove the transfer-related residues. Metallic contacts were prepared using e-beam lithography (Raith EBPG 5000+, 100 keV thermal field emission gun with an e-beam dose of ~950 µC/cm^2^) and e-beam evaporation of Pd (Alliance-Concept EVA 760, 80-nm thick). The deposition rate of Pd is 1 Å/s under the base pressure of ~1 × 10^−6^ mbar.

### Transport measurements

Cryogenic measurements were performed in an ICE Oxford liquid helium continuous flow cryo-magnetic system with a base temperature of ~1.5 K. Source-drain currents were measured using a Keithley sourcemeter 2636B, while a Keithley sourcemeter 2400 was used for applying bias through the SiO_2_ gate dielectric. The source voltage was varying between 0 and 2 V, while the drain was grounded.

### Electron microscopy sample preparation and imaging

After the identification of 1–3 layers of PtSe_2_ on Si/SiO_2_ substrate using AFM, the substrate was spin-coated with a PMMA A2 polymer (1500 rpm, ~150-nm thick) and baked on a hot plate at 60 °C for 4 min. Then, PMMA surrounding the target PtSe_2_ was scratched and detached after mildly etching SiO_2_ using a dilute KOH solution (0.7%, ~30 min). The floating PMMA A2 film was picked up and rinsed several times in DI water. Finally, the film was aligned on Si_3_N_4_ TEM grids with the help of a micro-manipulator using a glass nanocapillary. Supporting PMMA film was removed by using the cleaning protocol discussed above. Resulting crystals on TEM grids were annealed at 250 °C for 6 h under high vacuum conditions (~1 × 10^−6^ mbar) to further remove the transfer-related residues. Here, we note that a very diluted KOH concentration is used to eliminate the unintentional creation of defects in PtSe_2_. This effective transfer process was previously utilized for the STEM imaging of graphene, MoS_2_, and WS_2_ crystals which have extremely low concentration of defects.

Scanning transmission electron microscopy (STEM) imaging experiments were conducted using an aberration-corrected (with double Cs corrector) FEI Titan Themis TEM 60–300 kV, equipped with Schottky X-FEG electron source and a monochromator to reduce the effect of chromatic aberrations. To minimize the effect of electron-beam-induced knock-on damage, a low acceleration voltage (80 kV) was used for all the experiments. The typical electron probe current was 20 pA. Images were acquired with Gatan high annular angular dark-field (HAADF) detector using 185-mm camera length which corresponds to a 49.5–198 mrad collection angle.

### TEM simulations

Multislice STEM image simulations were performed using the Dr. Probe software package^44^. The frozen phonon method was used with 30 variations per pixels and 64 displacements per atom. The resulting sampling rate was ~0.02 nm/pixel. The simulation parameters were chosen to be similar to the experimental conditions. The beam semi-convergence angle and HAADF detector angle was set to 21.2, 49.5–198 mrad, respectively. The source size was chosen to 0.05 nm and all the aberrations were set to zero. The result of STEM image simulation confirms that there is no degradation or structural modification of PtSe_2_ in the process of transfer (see Supplementary Note 9).

### First-principles calculations

Our calculations were performed under the gradient-corrected, spin-orbit inclusive density-functional theory formalism, as implemented in VASP^45,46^. We treated the exchange and correlation effects at the PBE level^47^, but additionally verified the robustness of our results against the choice of the density functional (see Supplementary Note 10). The energy cutoff for the plane wave basis set was set to 500 eV. The integration over the Brillouin zone was carried out with the equivalent of 12 × 12 × 1 **k**-points per unit cell. These parameters were chosen after extensive convergence tests. During the geometry optimizations, the in-plane lattice constants and interlayer spacing for the bilayer systems were constrained to their experimental values of 3.73 and 5.08 Å, respectively, while atomic coordinates were relaxed until each residual component on the Hellmann–Feynman forces was lower than 0.01 eV Å^−1^. We modeled defective mono and bilayer PtSe_2_ by introducing a single Pt vacancy defect in an otherwise pristine 4 × 4 supercell containing 48 and 96 atoms, respectively, as such models host a Pt vacancy concentration of 14 × 10^12^ cm^−2^, very close to our experimental estimate of ~9 × 10^12^ cm^−2^. In Supplementary Note 10, we additionally show that our conclusions are insensitive to the size of the supercell. A vacuum region of 15Å was introduced to separate the periodic images.

## Supplementary information

Supplementary Information

## Data Availability

The data that support the findings of this study are available from the corresponding author on reasonable request.
