# Supplementary material for: Probing magnetism in atomically thin semiconducting PtSe2
Source: Nat Commun. 2020 Sep 23;11:4806. doi: 10.1038/s41467-020-18521-6 (PMC7511911; doi:10.1038/s41467-020-18521-6)
Supplement: Supplementary file 1 — Supplementary Information [file 41467_2020_18521_MOESM1_ESM.pdf]

Supplementary Information for

“Probing magnetism in atomically thin semiconducting $PtSe_2$”

Avsar *et al.*

Supplementary Note 1. Observation of variable range hopping in $PtSe_2$ monolayer up to room temperature

To shed light on the mechanism of charge transport in monolayer $PtSe_2$, we study the temperature dependence of conductivity. Supplementary Figure 1a shows the Arrhenius plot of the recorded conductivity as a function of carrier concentration. Strong deviation of the data from linearity indicates that the electron transport cannot be fully explained by thermally activated transport. Our device is extremely insulating below 115 K as shown in Fig. 1d. Above this temperature range, it starts to be more conductive and ln (G) has a linear dependence on $T^{-1/3}$ up to room temperature which is the indication of 2D variable range hopping (VRH) conduction (Supplementary Figure 1b).[1–4] Similar transport characteristics have been previously observed for several 2D crystals including MoS_2[1,3,4] and disordered graphene nanoribbons[5] and this indicates the presence of high concentration of defects which were directly imaged in our crystals by TEM as shown in Supplementary Figure 2 & 4.

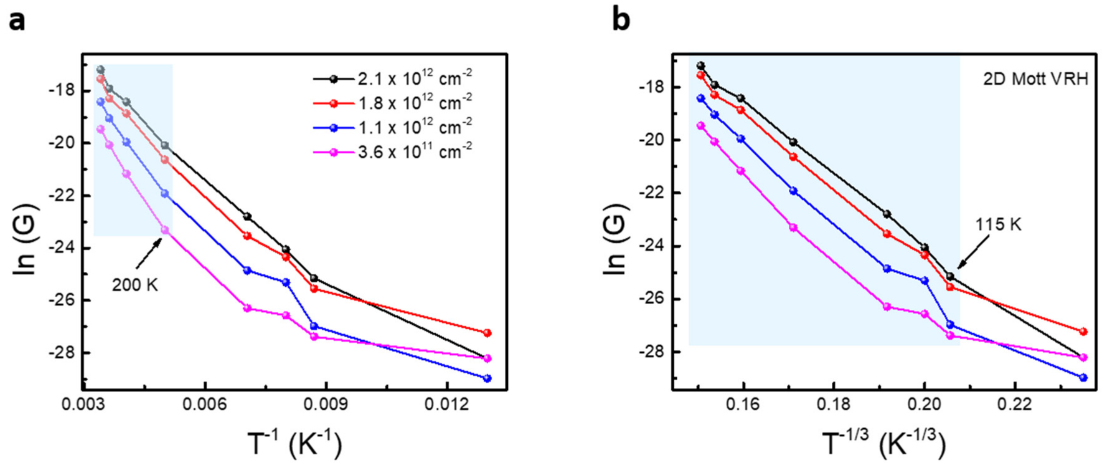

Supplementary Figure 1. **a,** ln (G) plotted against inverse of temperature for the monolayer $PtSe_2$ device. **b,** ln (G) plotted against $T^{-1/3}$ for the same device. Shaded areas represent the linear regimes.

Supplementary Note 2. Estimation of defect concentration in bilayer $PtSe_2$

Due to the ADF-STEM contrast dependency, the intensity of the atomic columns is proportional to the atomic number. Thus, the position of Pt (brighter) and Se (slightly darker) atoms can be directly interpreted by their intensities. Supplementary Figure 2a shows the clean large area of bilayer $PtSe_2$ lattice. To avoid the e-beam induced damage, a low beam current (18 pA) was used. Total e-beam doses for Supplementary Figure 2 a-c are 0.7×10^7, 0.33×10^7 and 0.17×10^7 e per nm^2, respectively. However, it varies to 2.8×10^7 e per nm^2 for the Fig. 1b in main manuscript. The magnified images from the same regions shows the atomic defect structures in the lattice.

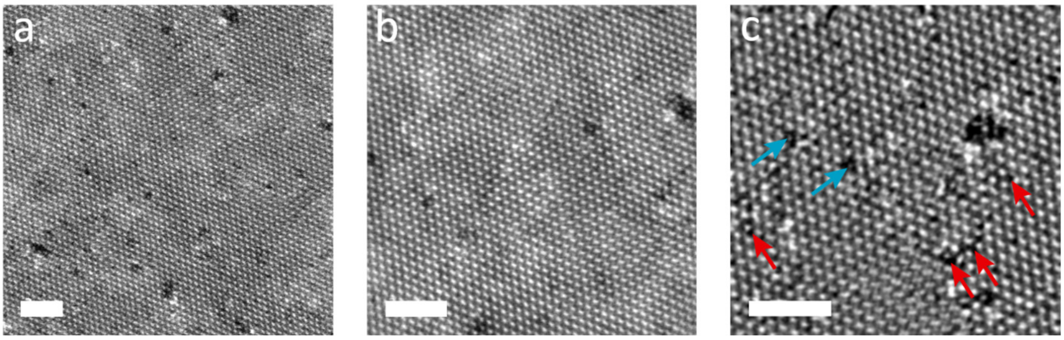

Supplementary Figure 2. STEM image acquired in a bilayer $PtSe_2$. Scanned area magnifies as it goes from a-to-c. Some of Pt (Se) point defects are highlighted with blue (red) colored arrows in c. Scale bars in a-c are 2 µm.

To measure the defect concentration, total number of missing Pt and Se atoms were counted directly using ImageJ. Total number of missing Pt and Se atoms in bilayer $PtSe_2$ were 79 and 278, respectively. Total measured area was 1095.81 nm^2. This corresponds to the Pt defect density $(0.9 \pm 0.06) \times 10^{13}$ cm^{-2}, whereas the Se defect concentration is $(3.5 \pm 0.2) \times 10^{13}$ cm^{-2}. Frequency distribution histograms for both Pt and Se are shown in Supplementary Figure 3. Here, we would like to also note that we have not observed any intercalates or crystalline phases other than the 1T phase.

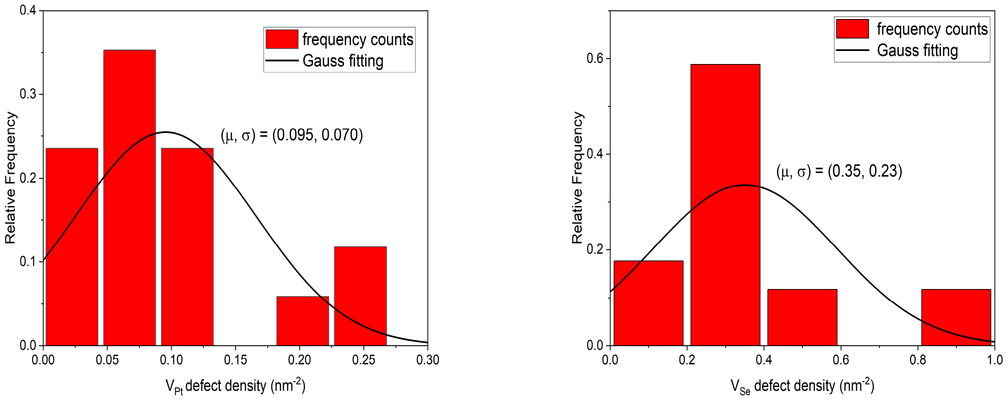

Supplementary Figure 3. Frequency distribution histograms for both Pt and Se vacancies.

Supplementary Note 3. TEM investigation of monolayer $PtSe_2$

Supplementary Figure 4 shows the STEM image of monolayer $PtSe_2$. As in the bilayer $PtSe_2$ case (Fig. 1b and Supplementary Figure 2), both Pt and Se point defects exist in monolayer $PtSe_2$. Total e-beam dose for Supplementary Figure 4 is 2.7×10^7 e per nm^2

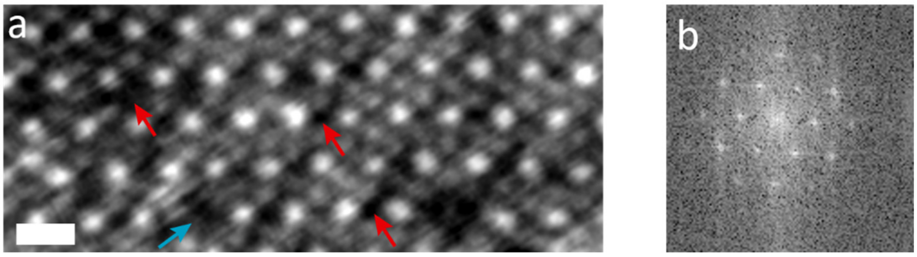

Supplementary Figure 4. (a) STEM image acquired in a monolayer $PtSe_2$. Some of Pt (Se) point defects are highlighted with blue (red) colored arrows. Scale bar is 1 µm. (b) Corresponding fast Fourier transform shows its crystalline structure.

Supplementary Note 4. Hysteresis in monolayer $PtSe_2$-based devices

We performed the direction dependent magneto-transport measurements on monolayer $PtSe_2$ (see Supplementary Figure 5b). Unlike metallic devices where we observe a small hysteresis as shown in Supplementary Figure 5a, we do not observe hysteresis in this semiconducting device, most likely due to higher noise to signal ratio compared to metallic devices. To double check this, we have fabricated and characterized an additional monolayer $PtSe_2$-based tunneling device in which the magnetic response was probed measuring the adjacent graphene layer as in Fig. 3c (Supplementary Figure 5c). While we observe a similar magneto-transport response with two clear plateaus, this device also shows little to no hysteresis. This could be related to the absence of net perpendicular anisotropy as our ribbon-type crystals have an in-plane spin orientation due to its shape anisotropy which could be compensating the magneto-crystalline anisotropy of the crystal that normally favors an out-of-plane spin orientation.[6]

We would like to note that hysteresis in bilayer CrI_3 which has similarly antiferromagnetic ground state ordering is also challenging to observe below the Neel temperature, e.g. it was absent in previous magneto-optical Kerr effect microscopy measurements conducted at 15 K, much below its Curie temperature of ~ 61 K.[6,7] Detailed temperature dependent tunneling experiments also confirm that hysteresis can only be probed at low temperatures [see Supplementary Figure 4 of Ref. 8]. The origin of this remains poorly understood. Here, we would like to note that the Curie temperature of $PtSe_2$ (typically ~ 5 K[9]) is much smaller than CrI_3 (~ 61 K) and probing the hysteresis is therefore even more challenging.

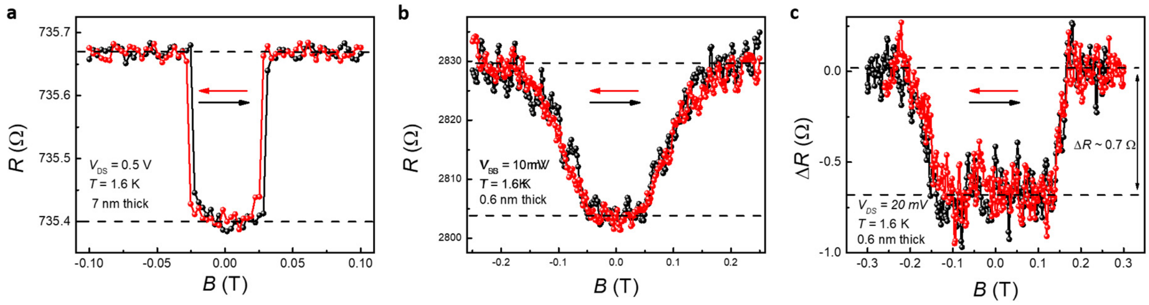

Supplementary Figure 5. Magnetic field dependence of the device resistances measured at *T*= 1.6 K, obtained from (a) a 7 nm thick $PtSe_2$-based lateral device, (b) a 0.6 nm thick $PtSe_2$-based tunneling device and (c) another 0.6 nm thick $PtSe_2$-based device. While lateral metallic device is contacted using Pd, tunneling devices utilize graphene (Pd) as a bottom (top) contact. Red and black arrows represent the magnetic field sweep directions.

Supplementary Note 5. Gate dependence of induced magnetization in graphene

Supplementary Figure 6 shows the back-gate voltage dependences of the device resistance and the change in the device resistance, Δ*R*, under magnetic field, measured in a monolayer graphene utilizing bilayer $PtSe_2$ as the substrate. Similarly to the device resistance, Δ*R* also shows the maximum value near the Dirac point of graphene. MR percentage is found to be ~0.1 % and is almost back-gate voltage independent.

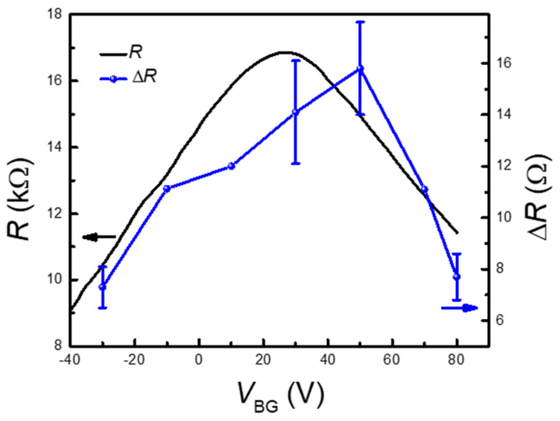

Supplementary Figure 6. Dependence of the change in device resistance and device resistance on the back-gate voltage at fixed bias of V_{SD} = 1 V. Measurements were taken at T = 1.6 K.

Supplementary Note 6. Background signal

For the device characterized in Fig. 3d, the background signal in these MR sweeps are direction dependent in contrast to our previous metallic devices (Supplementary Figure 7a-b).[9] While the origin of this is not clear at this point, this makes the successful demonstration of minor loop sweeps as in our previous work with metallic devices[9] nearly impossible as the amplitude of signal is comparable to the background level. This is also partly because the magnitude of the maximum change in device resistance in bilayer $PtSe_2$-based device is significantly smaller (by nearly a factor of 50) than what we observed in metallic samples.

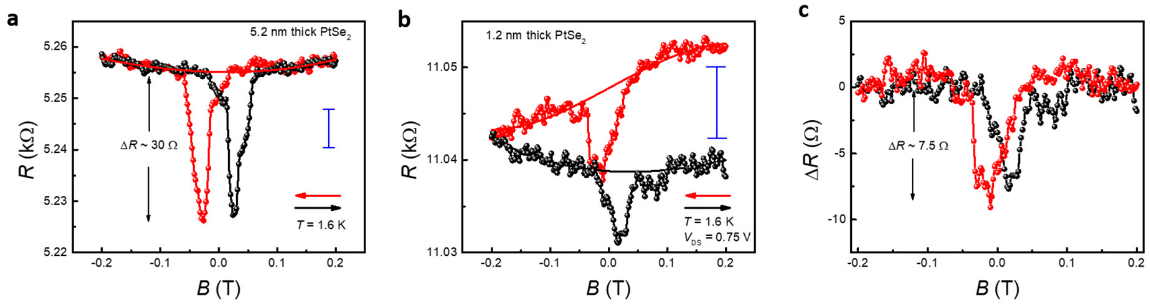

Supplementary Figure 7. Magnetic field dependence of the device resistances measured at T= 1.6 K, obtained from (a) a 5.2 nm thick $PtSe_2$-based lateral device, (b) a 1.2 nm thick $PtSe_2$-based tunneling device. Scale bars shown in these plots are 7.5 Ω. (c) The same with (b), after removing the background signal. Red and black arrows represent the field sweep direction.

Supplementary Note 7. Trace elemental analysis of $PtSe_2$

We studied the Inductively coupled plasma mass spectroscopy (ICP-MS) (NexIon 350 D instrument, Perkin Elmer) response of our CVT grown crystals for trace elemental analysis of $PtSe_2$. Two samples that were grown under different runs by the manufacturer (HQ graphene) were investigated (Supplementary Figure 8).

We first performed a quantitative assessment of our samples, which did not show the presence of any chemical elements except for Pt and Se. This unambiguously proves that our crystals are not contaminated with foreign elements, in line with our previous EDX[9] and TEM measurements. Furthermore, we would like to remark that the powder X-ray diffraction (XRD) provided by the manufacturer do not show any contamination by foreign atoms either. A copy of the XRD certificate from the manufacturer can be accessed at the Ref. 10. XRD 2 theta peak of (001) crystal plane of $PtSe_2$ is at 17.5degree, which matches well with the reported XRD analysis from other similarly grown $PtSe_2$.[11]

Next, we carried out a precise analysis to determine the mass ratios between Pt and Se atoms. To this end, calibration curves of platinum based on its isotope 195, and selenium based on its isotope 82 were obtained (See Supplementary Figure 8). The calculated mass (weight) Pt-to-Se ratio for sample 1 and 2 are 1.253 and 1.217, respectively which are closely matching the theoretical mass ratio of 1.235-to-1 for $PtSe_2$. To determine the errors of the measurement, we performed 5 repetitions on the same digested sample. Also note that each measurement by the instrument is already composed of triplicates, i.e. three individual passes. Calculated average mass ratio is 1.246 with a relative standard deviation of 1.4 %. Therefore, the difference between theory and experiment in percentage is only 0.84 %.

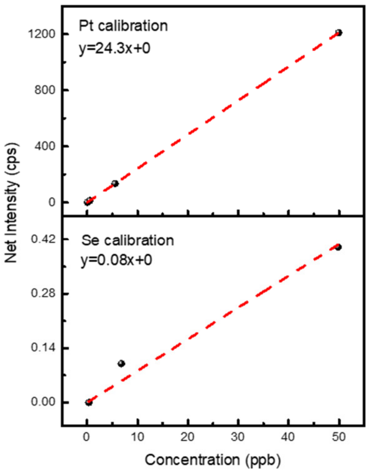

Sample id	Pt (ppb)	Se (ppb)	Mass ratio
Standard 1	0.05	0.05	
Standard 2	0.500	0.524	
Standard 3	4.999	5.026	
Standard 4	49.946	49.821	
Sample 1	212.108	169.320	1.252704
Sample 2	153.411	126.073	1.216835

Supplementary Figure 8. Bottom and top insets show the calibration curves for Selenium (Se) and Platinum (Pt). Table represents the concentration of Se and Pt that were measured in the solutions resulted from digestion. Calculated mass ratios for both samples are also provided in the table.

Supplementary Note 8. Raman spectroscopy measurements of ultra-thin $PtSe_2$

In order to determine the crystalline phase of our crystal, we have performed Raman spectroscopy measurements. Raman spectra show two different vibrational modes of comparable intensities at ~178 and 208 cm^{-1} (Supplementary Figure 9). These characteristic Raman peaks are assigned to E_g and A_{1g} modes respectively, with vibration modes illustrated in the inset of Supplementary Figure 9. The Raman response of our crystal is thus consistent with the one obtained from similar CVT-grown $PtSe_2$ which was later demonstrated to be 1T phase on the basis of direct TEM imaging measurements.[11,12] Our TEM studies also show the presence of 1T phase in our crystals, therefore consistent with the literature.

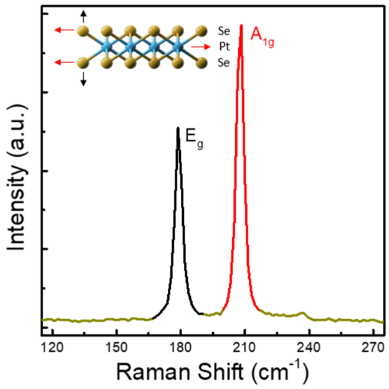

Supplementary Figure 9. Raman spectra of $PtSe_2$. Inset shows the crystal structure of monolayer $PtSe_2$. Red (black) arrows represent the oscillation direction of A_{1g} (E_g) phonons.

Supplementary Note 9. TEM simulation of bilayer $PtSe_2$

Prior to TEM scans, we perform AFM to determine the thickness of the investigated flakes. As mentioned in the Methods section, we use < 1% KOH solution to avoid or minimize any structural modification or degradation during sample transfer. To minimize the effect of electron-beam induced knock-on damage, a low acceleration voltage (80 kV) and a very short exposure time is used for all the experiments. To compare with the measured intensities across the dotted line shown in Fig. 1b, we performed STEM image simulations for a bilayer $PtSe_2$ by using the Dr. Probe software package.[13] The simulation parameters were chosen similar to the experimental conditions and details are given in the Methods section. As can be seen in Supplementary Figure 10, the line profile depicted in Fig. 1b with a single Pt vacancy can be fitted to this theoretical profile of electron transmission for bilayer $PtSe_2$.

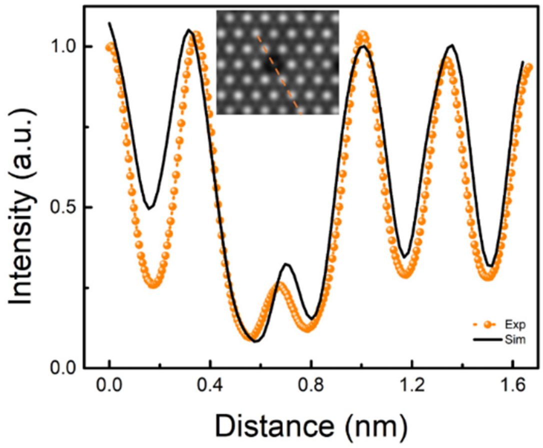

Supplementary Figure 10: Distance dependence of the intensity measured from a bilayer $PtSe_2$ and its simulated intensity. Inset shows the STEM image simulation for bilayer $PtSe_2$. Following the dashed orange line, we extract the simulated intensity.

Supplementary Note 10. Additional first-principles calculations

**Density Functional**	**E_{AFM} –E_{FM} (meV) (w/o SOC)**	**E_{AFM} –E_{FM} (meV) (w SOC)**
LDA	-42	-32
PBE	-29	-35
PBEsol	-3	-7
PW91	-63	-65
revPBE	-34	-44

Supplementary Table 1. Difference in energy between the antiferromagnetic (AFM) and ferromagnetic (FM) configurations for a 4 × 4 supercell of monolayer $PtSe_2$ hosting a Pt vacancy defect, as calculated using different local (LDA[14]) and semi-local (PBE,[15] PBEsol,[16] PW91,[17] revPBE[18]) exchange-correlation density functionals, either without and with spin-orbit coupling (SOC). Irrespective of the adopted functional, the AFM configuration is found to be the stable solution. In addition, the metastable FM configuration exhibits a local magnetic moment of 4.00 μ_B, in contrast to the findings of Ref. [19].

**Density Functional**	**E_{AFM} – E_{NM} (meV)**
LDA	-8
PBE	-89
PBEsol	-64
PW91	-27
revPBE	-94

Supplementary Table 2. Difference in energy between the antiferromagnetic (AFM) and non-magnetic (NM) configurations for a 4 × 4 supercell of monolayer $PtSe_2$ hosting a Pt vacancy defect, as calculated using different local (LDA[14]) and semi-local (PBE,[15] PBEsol,[16] PW91,[17] revPBE[18]) exchange-correlation density functionals. Irrespective of the adopted functional, the AFM configuration is found to be lower in energy as compared to the NM configuration.

**Supercell Size**	**E_{AFM} –E_{FM} (meV) (w/o SOC)**	**E_{AFM} –E_{FM} (meV) (w SOC)**
3 × 3	-8	-14
4 × 4	-29	-35
5 × 5	-43	-47
6 × 6	-44	-50

Supplementary Table 3. Difference in energy between the antiferromagnetic (AFM) and ferromagnetic (FM) configurations for monolayer $PtSe_2$ hosting a Pt vacancy defect for different supercell sizes, as calculated using the PBE exchange-correlation functional,[15] either without and with spin-orbit coupling (SOC). Irrespective of the supercell size, the AFM configuration is found to be the stable solution.

Supplementary References

1. Xue, J., Huang, S., Wang, J.-Y. & Xu, H. Q. Mott variable-range hopping transport in a MoS_2 nanoflake. *RSC Adv.* **9**, 17885–17890 (2019).
2. Mott, N. F. Conduction in non-crystalline materials. *The Philosophical Magazine: A Journal of Theoretical Experimental and Applied Physics* **19**, 835–852 (1969).
3. Qiu, H. *et al.* Hopping transport through defect-induced localized states in molybdenum disulphide. *Nature Communications* **4**, 2642 (2013).
4. Radisavljevic, B. & Kis, A. Mobility engineering and a metal-insulator transition in monolayer MoS_2. *Nature Materials* **12**, 815–820 (2013).
5. Han, M. Y., Brant, J. C. & Kim, P. Electron Transport in Disordered Graphene Nanoribbons. *Phys. Rev. Lett.* **104**, 056801 (2010).
6. Huang, B. *et al.* Layer-dependent ferromagnetism in a van der Waals crystal down to the monolayer limit. *Nature* **546**, 270–273 (2017).
7. Huang, B. *et al.* Electrical control of 2D magnetism in bilayer CrI3. *Nature Nanotechnology* **13**, 544–548 (2018).
8. Song, T. *et al.* Giant tunneling magnetoresistance in spin-filter van der Waals heterostructures. *Science* **360**, 1214–1218 (2018).
9. Avsar, A. *et al.* Defect induced, layer-modulated magnetism in ultrathin metallic $PtSe_2$. *Nature Nanotechnology* **14**, 674–678 (2019).
10. *http://www.hqgraphene.com/PtSe2.php*.
11. Yu, X. *et al.* Atomically thin noble metal dichalcogenide: a broadband mid-infrared semiconductor. *Nature Communications* **9**, 1545 (2018).
12. O'Brien, M. *et al.* Raman characterization of platinum diselenide thin films. *2D Mater.* **3**, 021004 (2016).
13. Barthel, J. Dr. Probe: A software for high-resolution STEM image simulation. *Ultramicroscopy* **193**, 1–11 (2018).
14. Ceperley, D. M. & Alder, B. J. Ground State of the Electron Gas by a Stochastic Method. *Phys. Rev. Lett.* **45**, 566–569 (1980).
15. Perdew, J. P., Burke, K. & Ernzerhof, M. Generalized Gradient Approximation Made Simple. *Phys. Rev. Lett.* **77**, 3865–3868 (1996).
16. Perdew, J. P. *et al.* Restoring the Density-Gradient Expansion for Exchange in Solids and Surfaces. *Phys. Rev. Lett.* **100**, 136406 (2008).
17. Perdew, J. P. & Wang, Y. Accurate and simple analytic representation of the electron-gas correlation energy. *Phys. Rev. B* **45**, 13244–13249 (1992).
18. Zhang, Y. & Yang, W. Comment on ``Generalized Gradient Approximation Made Simple''. *Phys. Rev. Lett.* **80**, 890–890 (1998).
19. Gao, J. *et al.* Structure, Stability, and Kinetics of Vacancy Defects in Monolayer $PtSe_2$: A First-Principles Study. *ACS Omega* **2**, 8640–8648 (2017).
